# Notes on a Case of Aphasia, with Some Suggestions Towards a New Hypothesis in Regard to the Production of That Form of Paralysis

**Published:** 1867-01

**Authors:** Joseph G. Richardson

**Affiliations:** Union Springs, Cayuga county, N. Y.


ART. II—*Notes on a case of Aphasia, with some suggestions towards a new hypothesis in regard to the production of that form of Paralysis.* BY JOSEPH G. RICHARDSON, M. D., *Union Springs, Cayuga county, N. Y.*

Mrs. S. L., aged 51, a lady of nervous temperament and active habits who had been married for thirty-two years, and was the mother of four children, found herself one morning in April, 1859, upon awaking, unable to speak at first, but after some minutes articulated the words "can't talk," and from that moment gradually recovered her speech until in a week the inability had disappeared. In three days more, however, another and similar attack occurred, accompanied by partial hemiplegia of the right side, causing her to drag her foot when she attempted to walk, which she began to do after five or six weeks; during the time she was confined to her bed she was able to move both upper and lower extremity to some extent, but complained much of numbness affecting them; the muscular paralysis gradually decreased for a space of three years, as did also her inability to articulate words, but she never recovered the power of reading, writing or counting; there seemed to be no true paralysis of the organs of speech, for she told her friends that the reason she could not talk better was that she could not think of the words; when asked a question she would sometimes reply "yes," and sometimes "no," entirely irrespective of the facts of the case, and often would enunciate one answer after the other repeatedly. Upon one occasion when it became important to have her signature to a deed, it was obtained by placing before her a slip of paper upon which the words composing her name were written, when she copied them without difficulty, and it is very remarkable that this signature was no mere servile imitation, but her veritable autograph, with all the peculiarities of her own hand-writing, indicating that what she herself declared to be was in truth her sole imperfection, viz: that she could not remember the words.

In February, 1863, she had another attack similar to the second above mentioned, which confined her to her bed for about a month, during which time she complained much of numbness and suffered severe headache, but was able to move her arm and leg to some extent, and for nearly six months from this time she had, on an average, about every three weeks a slight attack of numbness, (affecting always the right side) accompanied by severe headache, which lasted from twelve to forty-eight hours and passed off under mild treatment.

On the 27th of June, 1865, after a long period of immunity from these seizures, she began to complain of the customary symptoms which at first did not appear to be more severe than usual, but gradually passed on into stupor and profound coma; after remaining in this state for several days she began to rally and regained partial consciou ness, but with her right arm and leg completely paralyzed; her condition improved slightly for about three weeks, but at the end of that time strength commenced to fail, and on the 6th of September death took place. At the autopsy made the morning after her decease we found on removing the calvarium a marked depression in the substance of the brain, which presented the appearance of having been excavated over a surface of about an inch in diameter immediately above the termination of the fissure of Sylvius, and partly involving the island of Reil; it was evidently the result of the absorption of an apoplectic clot whose place was filled in part by a very loose cellular tissue containing some serous fluid in its folds. The anterior margin of this indentation was about 2½ inches from the anterior corner of the Centrum ovale majus, on the same side, measured upon the surface of the convolutions, and the distance of its superior margin from the edge of the great lougitudinal fissure on the same side was about 2¼ inches. Further incisions into the substance of the brain exhibited no evidence of softening, although many of the arteries at the base were calcified, especially those composing the circle of

Willis. In the left thalamus optici, however, there was a recent clot about the size of a small pea, which had undoubtedly been the cause of her last fatal attack.

Examined under the microscope with a power of 325 diam. I found the tissue filling the place of the old clot to be composed of fibrillœ, sometimes interlacing and sometimes par llel formed into bands from .005 to .01 of a millinetre in diameter, and entangling some compound granules and a few crystals of hematoidine. The surrounding structure was nearly healthy, only a few compound granules being observed scattered through the normal tissue. The clot in the thalamus optici and the portions of circumjacent, brain presented the usual microscopical appearances.

The theory of that distinguished French academician, M. Broca, who locates the group of mental faculties which Aphasia involves in the third frontal convolution of the left side, and that of Dr. Dax who circumscribes the localization still more closely, both maintain that the nerve centre presiding over these functions is single, and has its seat in the left hemisphere of the cerebrum, without any corresponding organ in the right side of the brain, but these suppositions although partly endorsed by even such high authorities as the late lamented M. Trosseau, have been recently contested by Prof. Austin Flint of Bellevue Hospital, New York, and are, I believe, still regarded by a majority of the profession as *sub judice*, being so contrary to the analogy which holds almost without exception in other portions of the human frame.

Mr. James Paget in that inexhaustable mine of thoughtful research and deduction, his work on Surgical Pathology, suggests that every idea which we receive is recognized by its producing some infinitely small, but positive alteration in a portion of the brain substance, and that the faculty of remembering depends upon this molocular change being perpetuated through all the permutations of degeneration and repair in the cerebral tissue, where it has occurred so that the mind can at any time again discover it; the mental vision recognizing this cicatrix, as it were, upon the tablet of memory, just as the outward eye might recollect a scar upon the visage of a friend. If this be so, were it not more in accordance with the well known idiosyncracies of nature to conclude that a double organ, one part in the third frontal convolution of *each* hemisphere, exists for the reception of impressions, which in health supply objective phenomena to the powers lost in a true aphasia: and to suppose that these faculties need two separate and distinct impressions, like those of a Stereoscope picture which must be combined into a single image by an effort of the mind for their full perfection; in other words that as the sense of sight requires binocular vision in order to appreciate the qualities of relief and rotundity, so the faculty of memory requires the compound of a double impression to recognize the attributes of mutual dependence and relation in the constituent of numbers and words.

On this hypothesis it is evident that were either of these twin centres overpowered by the pressure of a coagulum that single lesion must necessarily destroy the integrity of the faculties lost in such cases as mine above detailed; and the circumstance that most of the autopsies reported have shown effusion into the convolution just referred to may be accounted for by the well known fact that the left side of the body is weaker and more susceptible to disease of almost any kind than is its fellow.

